# Supplementary material for: Benchmarking Thiolate-Driven Photoswitching of Cyanine Dyes
Source: J Phys Chem B. 2023 Jan 13;127(3):732–41. doi: 10.1021/acs.jpcb.2c06872 (PMC9884076; doi:10.1021/acs.jpcb.2c06872)
Supplement: Supplementary file 1 — jp2c06872_si_001.pdf [file jp2c06872_si_001.pdf]

Benchmarking Thiolate Driven Photoswitching of Cyanine Dyes

Lucas Herdly,[†] Peter W. Tinning,[†] Angéline Geiser,[‡] Holly Taylor,[‡] Gwyn W. Gould,[‡] and Sebastian van de Linde*,[†]

†*Department of Physics, SUPA, University of Strathclyde, Glasgow, G4 0NG, Scotland, UK*

‡*Strathclyde Institute of Pharmacy and Biomedical Sciences, University of Strathclyde, Glasgow, G4 0RE, Scotland, UK*

E-mail: s.vandelinde@strath.ac.uk

Supporting Information

Methods

SMLM data analysis. The analysis of photoswitching kinetics was performed as previously described.[1] Raw acquisitions were processed with rapidSTORM 3.3[2] and subsequently analysed with Fiji[3] and custom written ImageJ macros.[4] rapidSTORM localization files were loaded into Fiji with 10 nm pixel resolution for the reconstructed images. The image was subdivided into 7×7 ROIs. For the geometrical inspection of localizations originating from single-molecule photoswitching (localization pattern), masks were created through Gaussian smoothing with 1 px standard deviation and thresholding as described. Only localization patterns were selected where the corresponding masks had a minimum circularity of 0.8 and a pixel area between 3 to 300. Raw localizations within selected localization patterns were then analyzed with respect to the molecule's on- and off-time intervals. For each ROI on- and off-time histograms were generated and fit to an exponential function to obtain the characteristic lifetimes of on- and off-state, respectively (Fig. S1). Fitting was performed multiple times with an incremental increase of the bin size of 1 (on-time) or 50 frames (off-time) if bins $<\tau/2.5$ to allow for obtaining fits with high R^2. For the on-time analysis, we allowed our algorithm to tolerate a gap of four frames between consecutive localizations, which was empirically found through an incremental increase of the gap interval until saturation.[1]

The photon count of each spot was extracted from the 2D Gaussian fit of the localization software, which is known to underestimate the total number of photons detected,[5,6] but for single-molecule surfaces this mismatch can be considered constant throughout the data set. The spot brightness was determined as the median photon count of all localizations passing the selection within a single ROI. Maps as shown in Fig. 1 were generated with the obtained photoswitching values for each ROI. For the kinetic analysis as shown in Figs. S2 and S3, outliers or values where τ_{on} was undersampled were excluded from further analysis.[1] This could occur in high intensity ROIs and was to some extent necessary for higher MEA concentrations and pH values. Numeric values as summarized in Fig. 2c and Fig. S4 were obtained from data fits and averaged if more than one acquisition per condition was made.

Typically, several hundred thousands of localizations were obtained for a single acquisition after passing the selection process as described above (cf. Fig. S1). E.g., for 10 mM MEA at pH 6.5, 631k localization were analyzed with on average 12.9k ± 2.6k localizations per ROI (mean ± SD) as well as 5.7k and 18.4k locs/ROI as minimum and maximum, respectively. 357k (min: 1.3k, max: 10.1k locs/ROI), 336k (min: 2.0, max: 9.4k locs/ROI), and 167k localizations (min: 0.8, max: 5.1k locs/ROI) were obtained for 10 mM pH 7.4, 50 mM pH 8.0 and 100 mM pH 8.5, respectively.

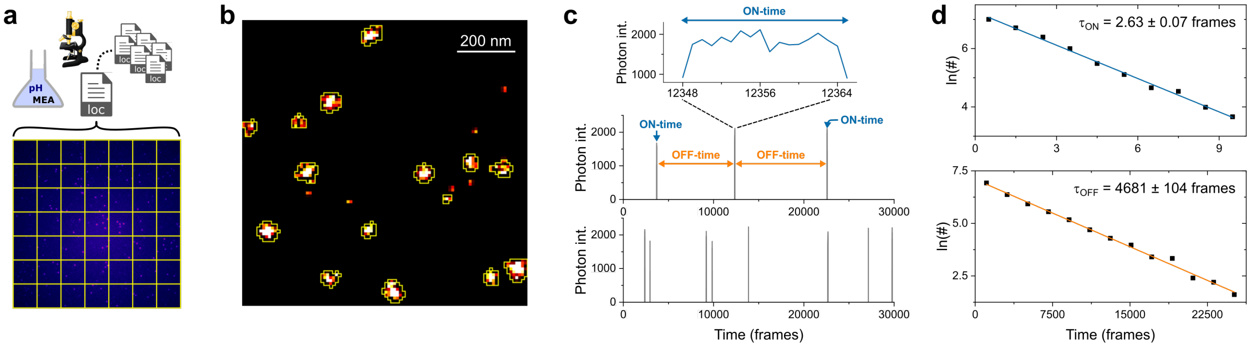

Figure S1: Single-molecule photoswitching analysis. **a**) For every buffer settings full FOV measurements of single-molecule surfaces were performed under dSTORM conditions (bottom). The obtained localizations were segmented into regions of interest (ROIs). **b**) Single localization patterns in the dSTORM image were subject to geometrical inspection; if successful (as indicated by the yellow selections) the corresponding localizations from the raw file were prepared as single-molecule time trace. The exemplary dSTORM image shows a small section of a single ROI. **c**) Time traces of each localization pattern were analyzed according to on- and off-time intervals, which were summed into an on-state and off-state histogram per ROI, respectively. **d**) The histograms were fitted to a single exponential decay using the function $\ln y = \ln a - kx$, with k as rate constant and $1/k$ as the characteristic lifetime τ. One frame corresponds to 50 ms.

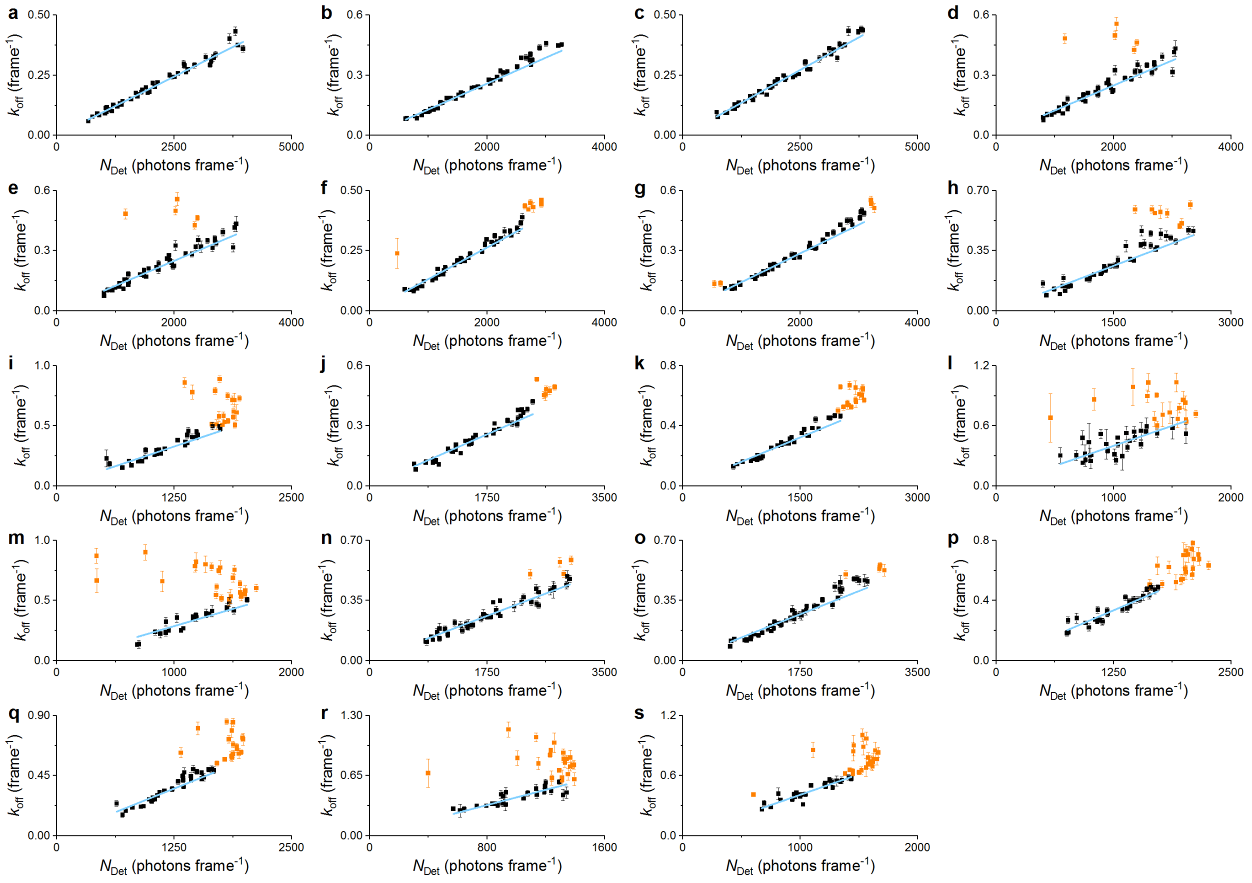

Figure S2: Linear correlation of k_{off} and N_{Det} for various buffer conditions. All buffer settings with enzymatic oxygen scavenger system. **a**) 10 mM MEA pH 6.5, **b, c**) 50 mM MEA pH 6.5, **d**) 100 mM MEA pH 6.5, **e**) 250 mM MEA pH 6.5, **f**) 10 mM MEA pH 7.4, **g**) 50 mM MEA pH 7.4, **h**) 100 mM MEA pH 7.4, **i**) 250 mM MEA pH 7.4, **j**) 10 mM MEA pH 8.0, **k**) 50 mM MEA pH 8.0, **l, m**) 100 mM MEA pH 8.0, **n, o**) 10 mM MEA pH 8.5, **p, q**) 50 mM MEA pH 8.5, **r, s**) 100 mM MEA pH 8.5. Fit function is shown as blue line. From the gradient of the fit the photon budget $N_{\tau\mathrm{on}}$ was determined. Extreme outliers and values subject to undersampling, shown in orange color, were excluded from analysis (cf. [1]). One frame corresponds to 50 ms. Error bars are standard errors from data fits.

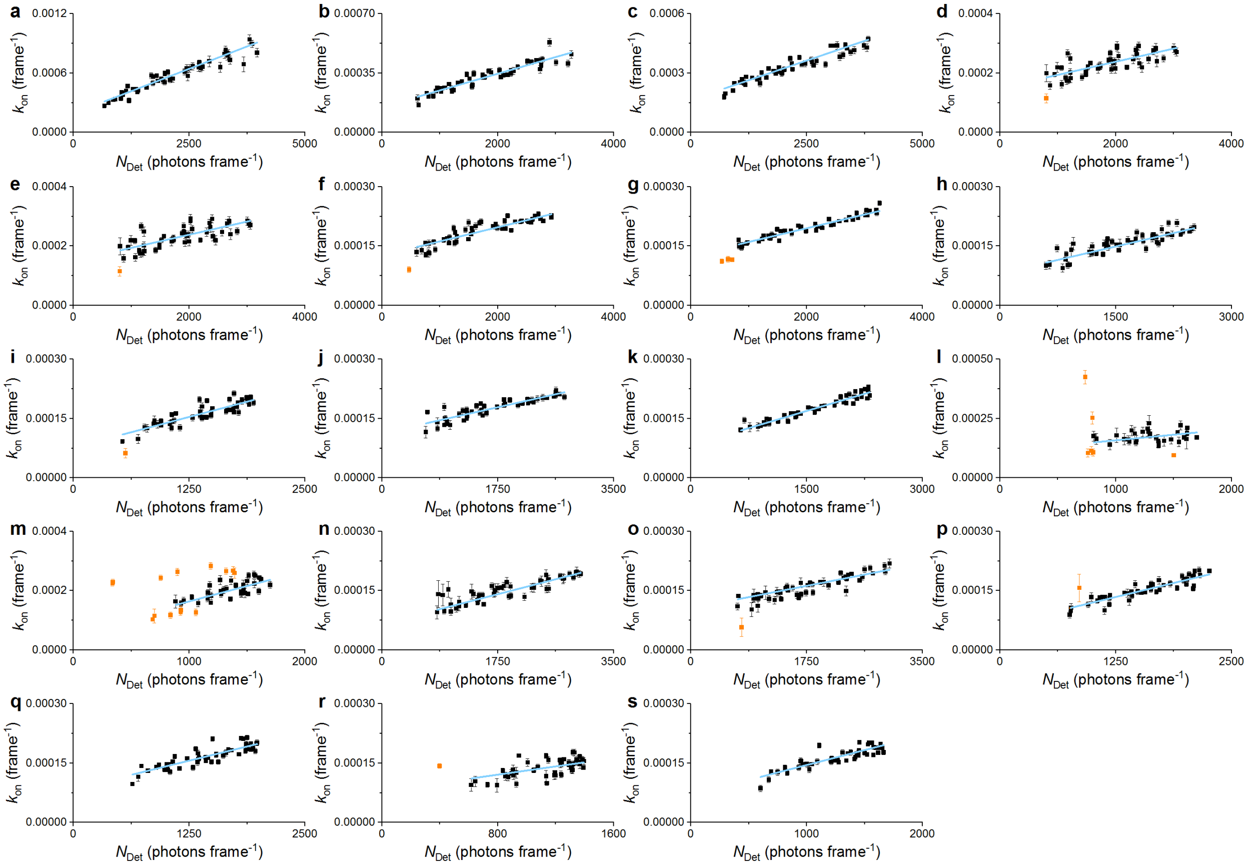

Figure S3: Linear correlation of k_{on} and N_{Det} for various buffer conditions. All buffer settings with enzymatic oxygen scavenger system. **a**) 10 mM MEA pH 6.5, **b, c**) 50 mM MEA pH 6.5, **d**) 100 mM MEA pH 6.5, **e**) 250 mM MEA pH 6.5, **f**) 10 mM MEA pH 7.4, **g**) 50 mM MEA pH 7.4, **h**) 100 mM MEA pH 7.4, **i**) 250 mM MEA pH 7.4, **j**) 10 mM MEA pH 8.0, **k**) 50 mM MEA pH 8.0, **l, m**) 100 mM MEA pH 8.0, **n, o**) 10 mM MEA pH 8.5, **p, q**) 50 mM MEA pH 8.5, **r, s**) 100 mM MEA pH 8.5. Fit function is shown as blue line. From the y-intercept the thermal dark state τ^0_{off} was determined. Extreme outliers and values subject to undersampling, shown in orange color, were excluded from analysis (cf. [1]). One frame corresponds to 50 ms. Error bars are standard errors from data fits.

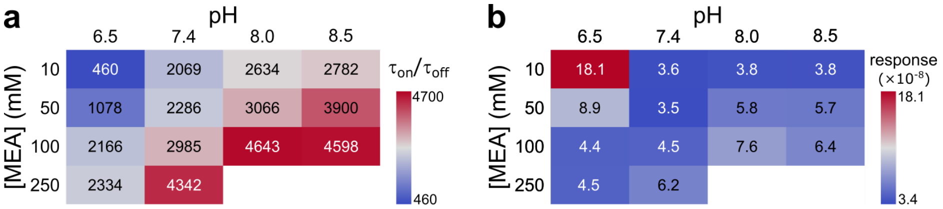

Figure S4: **a**) The maximum achievable $\tau_{\text{off}}/\tau_{\text{on}}$ in the FOV for different MEA concentrations and pH values. **b**) The response of k_{on} (frame^{-1}) on the spot brightness N_{Det} as determined from the gradient of the linear fit in Fig. 2b.

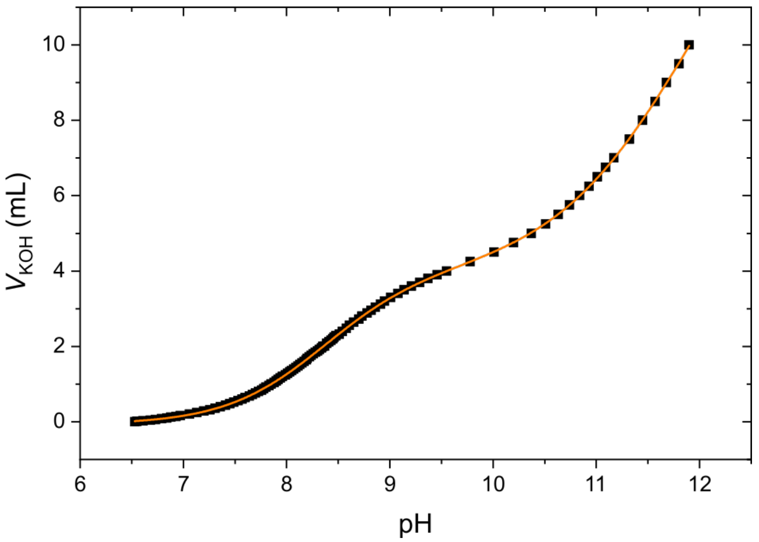

Figure S5: Titration of MEA. A solution of 100 mM mercaptoethylamine (MEA) hydrochloride was prepared in photoswitching buffer, i.e., 5% glucose, $10\,\mathrm{U\,mL^{-1}}$ glucose oxidase, $200\,\mathrm{U\,mL^{-1}}$ catalase, complemented with phosphate buffered saline, which was also used for the single-molecule imaging experiments in this work. Titration was carried out with 1 M potassium hydroxide (KOH). A sum of two Boltzmann functions was fit to the data to determine the pK_a of the thiol and amino group to pK_a1 = 8.353 ± 0.004 and pK_a2 11.931 ± 0.033, respectively (R^2 = 0.99998); $V = V_\mathrm{min} + V_\mathrm{max}(p(1+\exp(\frac{\mathrm{pH}-\mathrm{p}K_\mathrm{a1}}{k_1}))^{-1} + (1-p)(1+\exp(\frac{\mathrm{pH}-\mathrm{p}K_\mathrm{a2}}{k_2}))^{-1})$. The fit curve is shown as orange line.

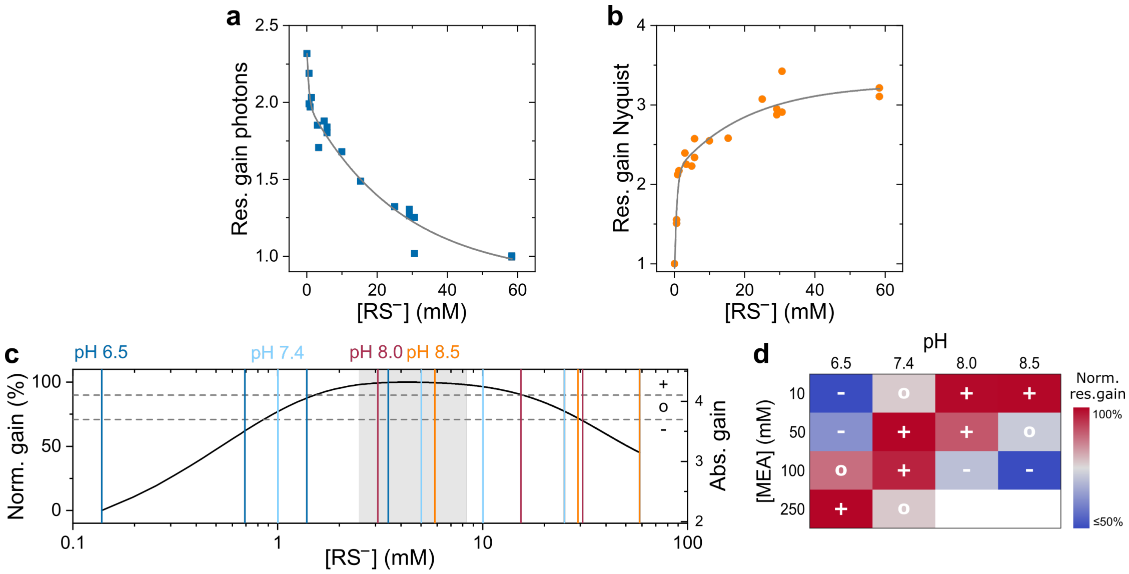

Figure S6: Optimal thiolate concentration. **a**, **b**) Resolution gain as shown in Fig. 4 but with linear scaling; **a**) resolution as determined by the photon limited localization precision and **b**) Nyquist resolution as determined by the maximum achievable label density, which is defined by the τ_{off}/τ_{on} ratio. Note the pronounced increase in Nyquist resolution toward 1 mM RS^-. **c**) Overall gain in resolution as determined by the product of the fit curves as shown in a) and b); left axis shows normalized, right axis absolute gain. Lower horizontal and upper dashed lines indicate 70.7% (-3 dB) and 90% of the total gain, respectively. A working range for the thiolate concentration can be determined between 1.5 and 15.6 mM with 90% total gain, the optimal range can be found between 2.5 and 8.3 mM (98%, light gray area) with 4.3 mM as maximum gain. The concentration bandwidth as determined at -3 dB allows to identify the lowest and highest RS^- concentration of 0.85 mM and 30.15 mM, respectively. The vertical lines indicate the tested buffer conditions; color indicates the pH with the MEA concentrations increasing from left to right: 10, 50, 100, (250) mM MEA. Buffer settings below 70.7% are marked $-$, between 70.7% and 90% $\circ$ and above 90% $+$ in panel d). **d**) Rating of the tested buffer conditions on the basis of the classification as shown in panel c).

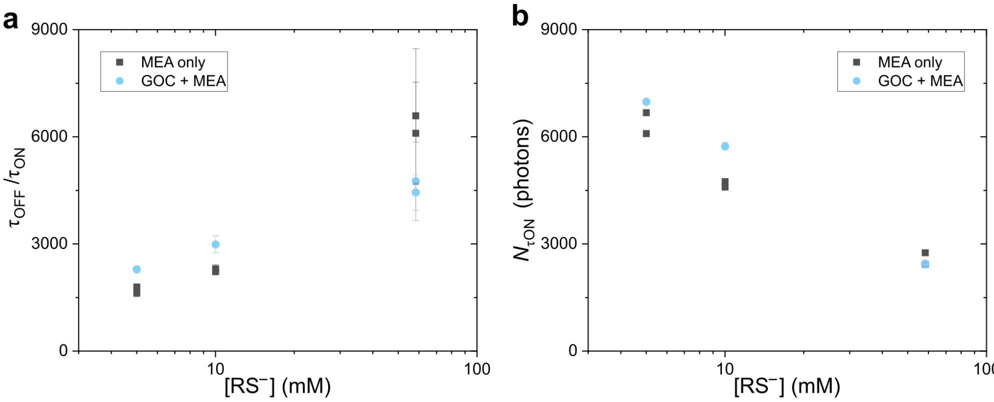

Figure S7: Photoswitching metrics of AF647 with and without enzymatic oxygen scavenger system. **a**) $\tau_{\mathrm{off}}/\tau_{\mathrm{on}}$ ratio and **b**) photon budget $N_{\tau\mathrm{on}}$ as function of the thiolate concentration.

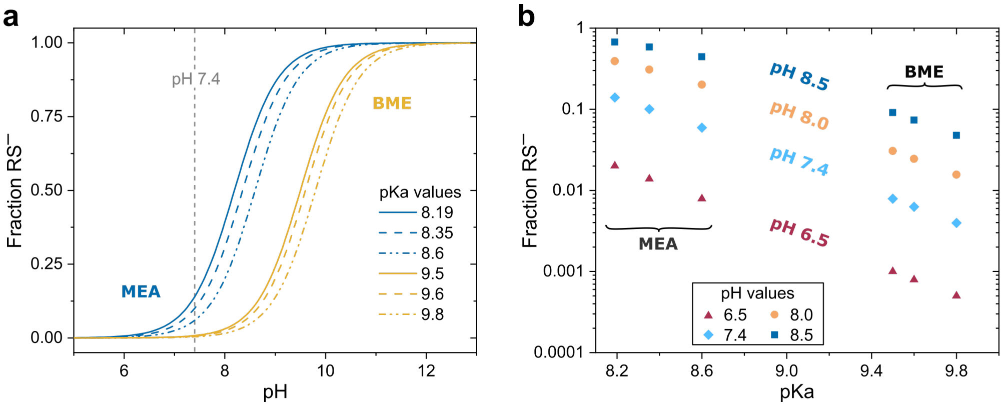

Figure S8: pK_a dependent fraction of thiolate RS^-. **a**) Fraction of RS^- for different pK_a values as a function of the pH. Curves in blue and orange show typical pK_a values as published for MEA and BME, respectively. **b**) Fraction of RS^- for different pH values as a function of the pK_a. Data points between 8.2 and 8.6 refer to pK_a values as published for MEA, whereas data points between 9.5 and 9.8 refer to pK_a values as published for BME.

Table S1: pH dependent thiolate concentration for varying MEA concentrations. All concentrations in mM. Thiolate concentrations were determined according to the Henderson-Hasselbalch equation (cf. Eq. (1)) with the pK_a as determined in Fig. S5.

**[MEA] (mM)**	**pH** 6.5	7.4	8.0	8.5
10	0.14	1.00	3.07	5.84
50	0.69	5.01	15.36	29.19
100	1.38	10.03	30.73	58.38
250	3.46	25.06	76.82	145.96

Table S2: Exemplary pK_a values as published for MEA and BME.

	**pK_a**	**Ref.**
**MEA**	8.19	Serjeant and Dempsey [7]
	8.27	Lide [8]
	8.31	Avdeef and Brown [9]
	8.35	Benesch and Benesch [10], Li and Manning [11], this work
	8.4	Suwandaratne et al. [12]
	8.6	Lundblad and Macdonald [13]
**BME**	9.5	Lundblad and Macdonald [13]
	9.61	Jencks and Salvesen [14]
	9.72	Serjeant and Dempsey [7], Lide [8]
	9.8	Suwandaratne et al. [12]

References

(1) Herdly, L.; Janin, P.; Bauer, R.; van de Linde, S. Tunable Wide-Field Illumination and Single-Molecule Photoswitching with a Single MEMS Mirror. *ACS Photonics* **2021**, *8*, 2728–2736.

(2) Wolter, S.; Löschberger, A.; Holm, T.; Aufmkolk, S.; Dabauvalle, M. C.; van de Linde, S.; Sauer, M. rapidSTORM: accurate, fast open-source software for localization microscopy. *Nat. Methods* **2012**, *9*, 1040–1.

(3) Schindelin, J.; Arganda-Carreras, I.; Frise, E.; Kaynig, V.; Longair, M.; Pietzsch, T.; Preibisch, S.; Rueden, C.; Saalfeld, S.; Schmid, B. et al. Fiji: an open-source platform for biological-image analysis. *Nat. Methods* **2012**, *9*, 676–82.

(4) van de Linde, S. Single-molecule localization microscopy analysis with ImageJ. *J. Phys. D: Appl. Phys.* **2019**, *52*, 203002.

(5) Mortensen, K. I.; Churchman, L. S.; Spudich, J. A.; Flyvbjerg, H. Optimized localization analysis for single-molecule tracking and super-resolution microscopy. *Nat. Methods* **2010**, *7*, 377–81.

(6) Franke, C.; Sauer, M.; van de Linde, S. Photometry unlocks 3D information from 2D localization microscopy data. *Nat. Methods* **2017**, *14*, 41–44.

(7) Serjeant, E. P.; Dempsey, B. *Ionisation constants of organic acids in aqueous solution*; IUPAC Chemical Data Series; no. 23; Pergamon Press: Oxford; New York, 1979.

(8) Lide, D. R., Ed. *Handbook of Chemistry and Physics*, 84th ed.; CRC Press: Boca Raton, 2003.

(9) Avdeef, A.; Brown, J. A. Cadmium binding by biological ligands. 2 [1]. Formation of protonated and polynuclear complexes between cadmium and 2-mercaptoethylamine. *Inorg. Chim. Acta* **1984**, *91*, 67–73.

(10) Benesch, R. E.; Benesch, R. The Acid Strength of the -SH Group in Cysteine and Related Compounds. *J. Am. Chem. Soc.* **1955**, *77*, 5877–5881.

(11) Li, N. C.; Manning, R. A. Some Metal Complexes of Sulfur-containing Amino Acids. *J. Am. Chem. Soc.* **1955**, *77*, 5225–5228.

(12) Suwandaratne, N.; Hu, J.; Siriwardana, K.; Gadogbe, M.; Zhang, D. Evaluation of Thiol Raman Activities and pKa Values Using Internally Referenced Raman-Based pH Titration. *Anal. Chem.* **2016**, *88*, 3624–3631.

(13) Lundblad, R. L., Macdonald, F., Eds. *Handbook of Biochemistry and Molecular Biology*, 5th ed.; CRC Press: Boca Raton, 2018.

(14) Jencks, W. P.; Salvesen, K. Equilibrium deuterium isotope effects on the ionization of thiol acids. *J. Am. Chem. Soc.* **1971**, *93*, 4433–4436.
